# Patient and healthcare provider perceptions on using patient-reported experience measures (PREMs) in routine clinical care: a systematic review of qualitative studies

**DOI:** 10.1186/s41687-022-00524-0

**Published:** 2022-12-02

**Authors:** Chindhu Shunmuga Sundaram, Rachel Campbell, Angela Ju, Madeleine T. King, Claudia Rutherford

**Affiliations:** 1 Faculty of Science, School of Psychology, Sydney Quality of Life Office, The University of Sydney, Level 6 North, Chris O’Brien Lifehouse (C39Z), Sydney, NSW 2006 Australia; 2 Faculty of Medicine and Health, The University of Sydney Susan Wakil School of Nursing and Midwifery, Cancer Care Research Unit (CCRU), The University of Sydney, Sydney, Australia; 3 The Daffodil Centre, The University of Sydney, a Joint Venture with Cancer Council NSW, Sydney, Australia

## Abstract

**Background:**

Patient-reported experience measures (PREMs) assess quality-of-care from patients’ perspectives. PREMs can be used to enhance patient-centered care and facilitate patient engagement in care. With increasing quality improvement studies in clinical practice, the use of PREMs has surged. As a result, knowledge about stakeholder experiences of using PREMs to assess quality of care across diverse clinical settings is needed to inform PREM implementation efforts. To address this, this review examines the qualitative literature on patient and healthcare provider experiences of using PREMs in clinical practice.

**Methods:**

Medline, Embase and PsycInfo were systematically searched from inception to May 2021. Additional searching of reference lists for all included articles and relevant review articles were performed. Retrieved articles were screened for eligibility by one reviewer and 25% cross-checked by a second reviewer across all stages of the review. Full texts meeting eligibility criteria were appraised against the COREQ checklist for quality assessment and thematic analysis was used to analyze textual data extracted from the results.

**Results:**

Electronic searches identified 2683 records, of which 20 studies met eligibility criteria. Extracted data were synthesized into six themes: facilitators to PREM implementation; barriers to PREM implementation; healthcare providers’ perspectives towards using PREMs; patients’ perspectives towards using PREMs; advantages of using PREMs in clinical practice; limitations and practical considerations to reduce resistance of PREM usage. The primary factors facilitating and impeding the use of PREMs include organizational-, staff- and patient-related factors.

**Conclusion:**

Results can be used to guide the usage and implementation of PREMs in clinical settings by addressing the identified barriers and building on the perceived benefits to encourage adoption of PREMs. Results around facilitators to PREM implementation and practical considerations could also promote appropriate use of PREMs by healthcare providers, helping to improve practice and the quality of care based on patient feedback.

**Supplementary Information:**

The online version contains supplementary material available at 10.1186/s41687-022-00524-0.

## Introduction

Assessment of patient experiences can improve the quality, experience, and outcomes of healthcare, and provide useful information for decisions about patient management and health service delivery [1–3]. Patient-reported experience measures (PREMs)—defined as ‘a measure of a patient’s perception of their personal experience of the healthcare they have received’ [2, 4, 5]—aim to identify where improvements in patient experience are required, judge the success of efforts to improve health services, and meaningfully capture what happens during the patient’s course of illness and treatment [3, 6].

Several generic and condition-specific PREMs have been developed and are currently used in clinical settings such as hospitals [3, 7]. The primary uses of PREMs include: (a) enabling patients to reflect comprehensively on interpersonal aspects of their healthcare experience; (b) providing reliable metrics for public reporting, benchmarking of institutions/centers and healthcare plans; and (c) generating patient-level information for driving service quality improvement strategies [1].

Despite these potential benefits, evidence is mixed regarding whether routine PREM assessment in clinical practice improves healthcare services [8–12]. Inconsistent findings may be due to lack of clear guidance on how to use PREM data to meaningfully inform improvements to the quality of care [13, 14]. Other possible challenges in their use include: (1) difficulty in measuring change at all levels of the healthcare system or institution [5, 10]; (2) time and resources available for staff to collect and analyze data [15]; (3) lack of consistency in measurement of patients’ experience across services and institutions [11]; (4) variation in how these data are used [11]; and (5) lack of skills and expertise among staff to effectively use and interpret patient experience data [16]. With limited resources, healthcare providers find it challenging to rigorously assess whether they do indeed provide cost effective and high-quality healthcare services.

Given the critical role healthcare professionals and patients play in ensuring PREMs data is used to successfully improve healthcare services, a better understanding of their experiences of using PREMs in routine clinical practice is needed. Perceptions of these key stakeholders may yield unique insight into how PREM programs could be implemented more effectively in clinical practice to guide meaningful improvements in care. This is important to ensure that the time and money invested in collecting PREM data is not wasted, and the benefits fully realized. To explore this issue in-depth, we conducted a systematic review of the qualitative literature to examine the perspectives of patients and healthcare providers towards using PREMS in routine clinical care.

## Methods

Our systematic review of qualitative studies was conducted according to the Preferred Reporting Items for Systematic Reviews and Meta-Analyses (PRISMA) guidance [17]. We limited our review to qualitative studies as these types of designs allow for in-depth enquiry into patient and healthcare provider perceptions of implementing PREMs in routine practice.

### Searches

Our search strategy comprised a comprehensive set of key terms for ‘PREMs’ and ‘qualitative research’ (Additional file 1: appendix 1). Searches were performed in electronic databases—MEDLINE, EMBASE, and PsycINFO from inception to May 2021. Electronic searches were supplemented by searches of reference lists of included studies and identified related papers.

### Eligibility criteria

Studies that met the following criteria were included:

- Qualitative study design (e.g. individual interviews or focus groups); mixed method studies were considered if a qualitative component was included;
- Sample was any patient and/or healthcare professional
- Focus of the study was on exploring perceptions of implementing patient-reported measures (PRMs) in routine clinical practice and the PRM focus was on experiences of healthcare

Studies were excluded if they were not in English; did not have adequate information (e.g. conference abstracts); or focused on PREM development, validation, or selection for use in a particular clinical setting.

### Study selection

One reviewer (AJ or RC) screened retrieved titles and abstracts for eligibility and 25% selected at random were cross-checked [18] by a second reviewer (CR). Where abstracts met eligibility or relevance was ambiguous, papers were obtained and reviewed in full. Full texts were independently reviewed by two reviewers (CS and AJ or RC). Disagreements were resolved through team discussion.

### Data extraction

A data extraction form was developed including study title, identifying information (author names, country), methods, aims, location and setting, study design, participants (population group, eligibility criteria, sample size, population characteristics), recruitment method, PREMs used, and study findings. All information from included studies were extracted by one reviewer (AJ) and cross checked by a second reviewer (CS) for accuracy against the original article.

### Quality assessment

Included studies were assessed for quality against the 32-item Consolidated Criteria for Reporting Qualitative research (COREQ) checklist [19] by one reviewer (AJ). A second reviewer (CS) assessed study quality for 25% of included studies. The COREQ checklist assesses reporting in qualitative literature in three domains: (1) research team and reflexivity (includes personal characteristics, relationship with participants); (2) study design (includes theoretical framework, participant selection, setting, data collection); and (3) data analysis and findings (includes data analysis, reporting). It includes 32 items and is scored as 0 = not reported, 1 = partially reported, and 2 = fully reported, with each article receiving a total quality score out of 64, converted into a percentage. Thus, higher scores indicate higher quality reporting.

### Synthesis of results

We used an interpretive descriptive approach using thematic analysis [20] to analyze the textual data extracted from the results sections of studies included in this review. This methodology was selected as it offers good transparency and is an adaptation of secondary data synthesis of ‘thematic analyses’. The process involved three stages: (1) becoming familiar with the data extracted from studies and generating initial codes, (2) identifying similarities between codes and searching for themes, (3) reviewing, and defining themes both within and across studies. Two reviewers (AJ and CS; both post-doctoral researchers with expertise in PRM methodology) independently coded from each study. Descriptive themes developed as findings from studies representing similar phenomena were grouped. These were then collated into higher level analytical themes according to our research questions. A third reviewer (CR; experienced PRM methodologist) reviewed the themes and codes to ensure that they accurately reflected the data reported in included studies.Hence, there was minimal potential for bias on data generation and analysis. Study characteristics were considered to explain any differences in findings across studies [20].

## Results

### Study selection

**Fig. 1:**
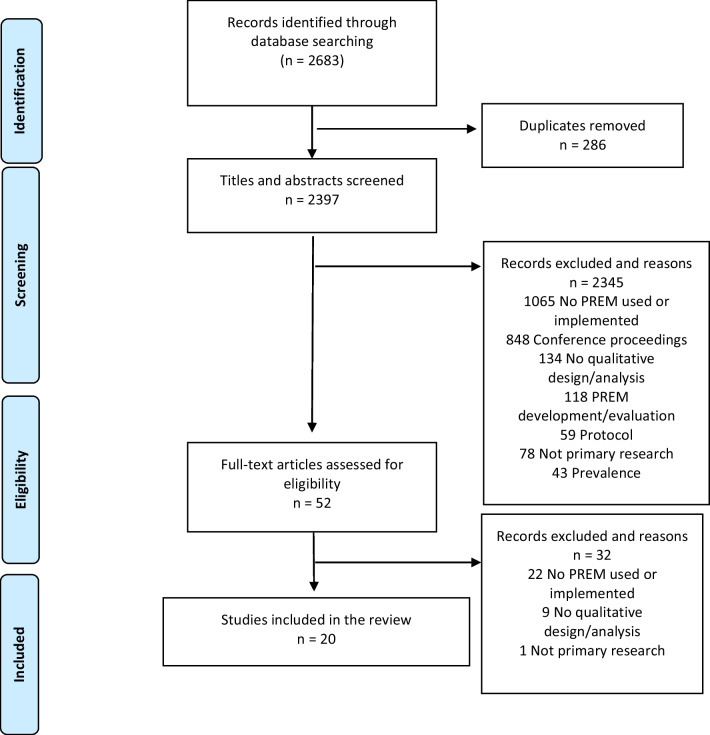
Prisma flow diagram describing study inclusion for the review

Electronic searches retrieved 2683 abstracts, of which 52 were potentially relevant and 20 articles met eligibility criteria (Fig. 1). Of these, nine used semi-structured interviews, two used focus groups, and nine used mixed methods (i.e., combination of semi-structured interviews, focus groups, patient surveys and/or clinician surveys) (Table 1).

**Table 1:** Summary of included studies reporting healthcare professionals’ and patients’ perspectives on using PREMs (N = 20)

References	Study aim	Study setting (clinical setting)	Patient population (sample size, disease/condition, gender m:f, age, ethnicity)	Clinician population (sample size, occupation, age, years in specialty)	Study design (method of data collection, method of data analysis)	Number of interviews/focus groups conducted	PREMs (PREM used, mode of administration, administration time points)
Barr et al. [30]	To explore the impact of state-wide public reporting of hospital patient satisfaction on hospital quality improvement (QI), using Rhode Island (RI) as a case example	General, inpatient rehab and psychiatric hospitals	Adult patients with an overnight stay who received medical, surgical or obstetrical services, and psychiatric patients. (Sample size, gender ratio, age, and ethnicity nr	42, CEO’s, medical directors, nurse executives, and patient satisfaction coordinators (age and years in specialty nr)	Mixed (Semi-structured interview and patient questionnaires)	Interviews: 42	Standardised state-wide patient satisfaction survey, hardcopy, after discharge
Boyce et al. [25]	To explore surgeon’s experiences of receiving peer benchmarked PROMs feedback and to examine whether this information led to changes in their practice	Hospitals	759, Hip replacement patients, (Gender, age and ethnicity nr)	11, Consultant orthopaedic surgeons, (age and years in specialty nr)	Mixed (Semi-structured interview and patient questionnaires)	Interviews: 11	Peer-benchmarked PRMs including OHS, EQ-5D, shortened version of HOOS and general health status item, hardcopy, before and 6 months post-surgery
Burt et al. [4]	To explore the views of primary care practice staff regarding the utility of patient experience surveys	Primary care (general practice)	N/A	127; 38 GPs, 19 practice managers, 18 nurses, 21 receptionists, 13 administrators, secretaries, and 18 other staff (dispensers and health-care assistants); (age and years in specialty nr)	Focus groups	Interviews: 127	Patient experience survey
Carter et al. [21]	To look at the ways in which general practices respond to patient feedback, both in terms of process and outcomes. It also considered consumers’ and primary care organisations’ suggestions and perspectives articulated during discussions about patient feedback	Primary care (general practice)	8600, general patients (Gender, age and ethnicity nr)	88, GP and primary care trust teams. (age and years in specialty nr)	Mixed (Focus groups and patient questionnaires), Interpretative Phenomenological Analysis (IPA)	Interviews: 88	IPQ, hardcopy, post-consultation
Tirado et al. [37]	To explore the benefits of, and challenges to, using PRMs for service quality improvement in clinical genetics, achieved through a case-study of a local clinician-led service quality improvement initiative	All Wales Medical Genomics Service (AWMGS)	(96, Patients attending AWMGS) (Gender, age and ethnicity nr)	6, Clinical genetics consultants, genetic counsellors, 1–25y; (years in specialty nr)	Mixed (interview and patient questionnaires), SPSS	Interviews: 6	EQ-5D, GCOS-24, AWMGS satisfaction questionnaire, hardcopy, before clinic attendance (EQ-5D, GCOS-24) or after clinic visit (AWMGS satisfaction questionnaire)
Davies et al. [8]	To develop a framework for understanding factors affecting the use of patient survey data in quality improvement	Health plans, medical groups and hospitals	No characteristics reported	14, Medical, clinical improvement, service quality directors, clinical improvement coordinators and managers, 2–20 years (team leaders only); (age and years in specialty nr)	Mixed (Semi-structured interviews and literature review), manually reviewing transcripts	Interviews: 14	nr
Davies et al. [27]	To evaluate the use of a modified CAHPS survey to support quality improvement in a collaborative focused on patient-centred care, assess subsequent changes in patient experiences, and identify factors that promoted or impeded data use	Health plans, medical groups and hospitals	General patients, (Sample size, gender, age and ethnicity nr)	50, Medical directors, directors of clinical improvement or service quality, group manager and quality improvement staff including team leaders, (age and years in specialty nr)	Mixed (Interview and survey) Quality Desktop TM	8 leaders identified They brought a total of 50 staff to attend meetings for collaborative activities Interviews: 7	CAHPS survey, telephone, before, after, and continuously over 12 months of the project
Davies et al. [35]	To assess factors that were barriers to, or promoters of, efforts to improve care experiences in VA facilities	Veterans’ Health Administrations	Surgical inpatients (Sample size, gender, age and ethnicity nr)	8, Executive director, patient advocates, customer services managers, ward nurse, and advanced nurse practitioner. (age and years in specialty nr)	Interviews, content analysis	Interviews: 8	SHEP, hardcopy, post-discharge
D'Lima et al. [26]	To report experience of anaesthetists participating in long-term initiative to provide comprehensive personalised feedback to consultants on patient-reported quality of recovery indicators	Hospital	Surgical patients in recovery (Sample size, gender, age and ethnicity nr)	21; 13 Consultant anaesthetists, 6 surgical nursing leads, theatre manager, clinical coordinator for recovery, nr, 2–32 years (Anaesthetists only) (years in specialty nr)	Semi-structured interviews, grounded theory	Interviews: 21	nr
Farrington et al. [22]	To explore doctors’ perceptions of patient experience surveys in primary and secondary care settings in order to deepen understandings of how doctors view the plausibility of such surveys	Primary and secondary care GP clinics	No sample characteristics reported	41, Primary (GP) and secondary (dermatology, gynaecology, neurosurgery, plastic surgery, renal medicine and rheumatology) doctors. (age and years in specialty nr)	Semi-structured interviews, NVivo	Interviews: 41	GPPS and National GMC patient questionnaire, hardcopy, after clinical consultation
Friedberg et al. [23]	To examine whether and how physician groups are using patient experience data to improve patient care	Primary care	No sample characteristics reported	Nr, leaders of physician groups including medical director, administrator or manager. (age and years in specialty nr)	Semi-structured interview, content analysis	nr	nr
Heineman et al. [38]	To describe the experiences of seven prosthetic clinics provided with external facilitation in collecting and sustaining patient-reported data collection as part of routine patient care and implementing QI activities	Prosthetic clinics	250, Prosthetics and orthotics patients, Prosthetics and orthotics patients, 155:95. Age, gender and ethnicity nr	Nr, Certified prosthetists, residents and others. (age and years in specialty nr)	Mixed (QI consultations/meetings and clinician and patient survey), ethnography	nr	OPUS, hardcopy, at admission, after device delivery and at 2-month follow-up
Lucock et al. [33]	To identify the barriers and facilitators to effective implementation and clinician engagement within a complex routine UK service setting	Psychological therapy services	197, Mental health, 70:132, 39, Gender and ethnicity nr	42, Psychological therapists, trainee clinical psychologists and temporary or short-term sessional therapists. (age and years in specialty nr) 26 permanent and qualified therapists, 8 trainee clinical psychologists, and 8 therapists either employed on a temporary basis to address the waiting list or who were based in another part of the service and provided short-term, sessional input. Of the 26 permanent and qualified therapists, 7 were cognitive behavioural therapists, 3 psychodynamic psychotherapists, and 16 clinical psychologists	Mixed (Patient outcome measures, therapist questionnaire, therapist review meetings, patient questionnaire, patient focus groups)	Focus groups: 2	CORE-10, ASC, HASQ, ARM-5, patient experience questionnaire, hardcopy, post-discharge (before session 5)
Reeves and Seccombe [28]	To assess current attitudes towards the national patient survey programme in England, establish the extent to which survey results are used and identify barriers and incentives for using them	Hospital	850 per NHS, General patients, Age, gender and ethnicity nr	24, Director of Nursing, Director of Patient and Public Involvement, Quality Development Manager, Head of Clinical Governance and others. (age and years in specialty nr)	Semi-structured interview, manually coded and categorised	Interviews: 24 (no patients)	NHS annual patient surveys including traffic light charts (administration time points nr)
Rooijen et al. [34]	To provide insight into experiences with the implementation and current ways of working with a patient-reported experience measure as an integrated measurement strategy	Disability care organisation	8, individuals with disability, Age, gender and ethnicity nr	Nr; quality manager, healthcare professionals trained in PREMs, trainers; (age and years in specialty nr)	Semi-structured interviews and focus groups	Interviews: 3 Focus group: 3 Manager, trainer, clients, researcher, quality manager Client interviews: 8	PREM used in the Dutch disability care sector called ‘How I Feel About It!’ (administration time points nr)
Scott et al. [29]	To determine the feasibility of implementing a patient safety survey which measures patients’ experiences of their own safety relating to a care transition	Hospital	28; cardiology, geriatrics, orthopaedics, stroke; Age, gender and ethnicity nr	21, ward sisters, discharge coordinators, ward receptionists, apprentices, nurses, patient safety leads, research nurses, occupational therapists, community matron; (age and years in specialty nr)	Mixed methods approach (Surveys, Interviews, focus groups and staff incident reports)	Patient interviews: 28 Staff interviews: 21	Patient safety survey (administration time points nr)
Alvarado et al. [36]	Exploring variation in the use of feedback from national clinical audits: a realist investigation	NHS Trusts (Three large teaching hospitals and two District General Hospitals)	None	54, doctors, nurses, audit support staff, trust board and committee members, quality and safety staff, information staff	Semi-structured interviews, NVivo	Staff interviews: 54	National Clinical Audit feedback (Administration nr)
Berger et al. [31]	Using patient feedback to drive quality improvement in hospitals: a qualitative study	Hospitals	None	9, Managers, supervisors and Director	Semi-structured interviews, NVivo, and document analysis	Staff interviews: 9	Patient feedback forms, data consolidation reports, action plans, process stands and protocols and institutional websites, social networks and service site for patient feedback/complaints. (No other details reported)
Squitieri et al. [32]	Patient-reported experience measures are essential to improving quality of care for chronic wounds: An international qualitative study	Wound centres in Canada, Denmark, The Netherlands and USA	60, wound patients, 35:25; Canada 12, Denmark 21, The Netherlands 15 and USA 12; Age nr	None	Semi-structured interviews, Interpretive decision approach	Patient interviews: 60	WOUND-Q
Siantz et al. [24]	Patient Experience with a Large-Scale Integrated Behavioral Health and Primary Care Initiative: A Qualitative Study	Community health settings	54 patients, chronic care condition and behavioural health condition; Age, gender, ethnicity nr	32, registered nurses, primary care providers, care coordinators and behavioural health specialists	Semi-structured interview, Focus groups	Patient Focus groups: 8 Staff interviews: 32	nr

m:f, male:female ratio; nr, not reported; N/A, not applicable; QI, quality improvement; PRMs, patient-reported measures; GP, general practitioners; USA, The United States of America; UK, The United Kingdom; NHS, National Health Service; OHS, Oxford Hip Score; EQ-5D European Quality of life Five-dimension; IPQ, Improving Practice Questionnaire; NVivo, qualitative data analysis computer software package produced by QSR International; SPSS, Statistical Package for Social Sciences; GCOS-24, Genetic Counselling Outcome Scale, 24; AWMGS, All Wales Medical Genetics Service Satisfaction questionnaire; HOOS, Hip Osteoarthritis and Outcome Score; GPPS, National General practitioner Patient Survey; OPUS, Orthotics Prosthetics Users’ Survey; CAHPS, Consumer Assessment of Healthcare Providers and Systems; VA, Veterans Health Administration; SHEP, Survey of Healthcare Experiences of Patients; CORE-10, Clinical Outcomes in Routine Evaluation, 10; ASC, The Assessment for Signal Cases; HASQ, Helpfulness Alliance and Stage Measure; ARM, Agnew relationship measure-5

### Study characteristics

Table 1 provides a summary of the 20 included studies. Five studies (25%) were conducted in primary care settings [4, 21–24], nine (45%) in hospitals [8, 25–32], and the rest either in psychological therapy services [33], a disability care organization [34] or other healthcare settings such as Veterans Health Administration [35] or National Health Service (NHS) [36]. Studies were conducted in the UK (N = 9; 45%), the USA (N = 8; 40%), Ireland (N = 1; 5%), Brazil (N = 1; 5%) and the Netherlands (N = 1; 5%). Sixteen (80%) included clinicians only, one (5%) included patients only, and three (15%) included both patients and clinicians. Total sample size across studies was 150 patients (range across studies = 3 to 127) and 553 clinicians (range across studies = 8 to 60). Patient participants were diverse, including chronic wounds, chronic conditions, adult cardiology, geriatric, orthopedic and stroke patients. Clinician participants included medical and executive directors, CEOs, surgeons, general practitioners, anesthetists, secondary care doctors, psychologists, counsellors, nurses, patient satisfaction coordinators, other allied health professionals, integrated care providers and administrative staff.

The main aims of included studies were quality improvement, exploration of benefits and challenges of using PREMs in clinical practice, assessing factors affecting use of PREM data in quality improvement, clinicians’ perceptions of the usefulness of PREM data, and investigating ways to effectively implement PREM data in routine clinical practice.

### Quality appraisal

**Fig. 2:**
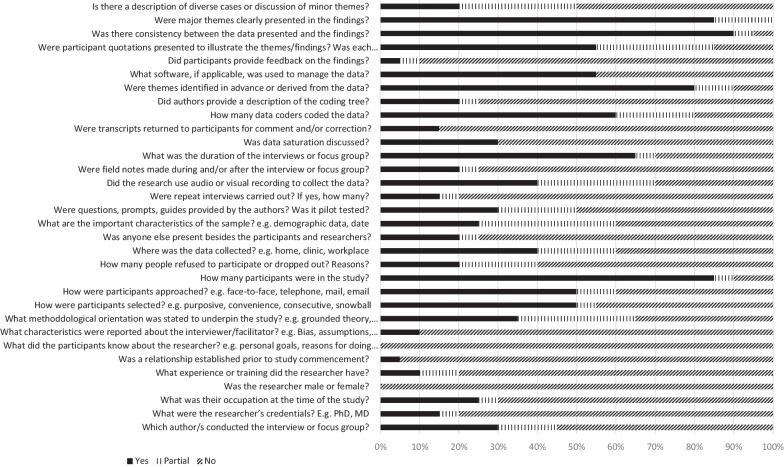
Quality of reporting across included articles (n = 20) per COREQ item

Study quality ranged from 20.3 to 57.8% [4, 21–29, 31–38]. No study met all quality criteria; however, some quality items were adequately addressed by all studies (Fig. 2). All studies clearly reported their major themes, 19 of 20 studies reported on the consistency between the data presented and study findings, and 18 of 20 studies reported on derivation of themes. However, most studies (84%) did not describe the research team and reflexivity in detail including interviewer characteristics, nature of the relationship with interviewees, and participants’ feedback on findings (Fig. 2).

### Synthesis of results

The findings across studies were synthesized into six themes: facilitators to PREM implementation; barriers to PREM implementation; healthcare providers’ perspectives towards using PREMs; patients’ perspectives towards using PREMs; advantages of using PREMs in clinical practice; limitations and practical considerations to reduce resistance of PREM usage.

#### Facilitators to PREM implementation

Studies reported PREMs were best used in a cyclical manner to give staff time to review findings and implement changes [25, 30, 33]. Organizational facilitators included a working culture supportive of improvement, change and recognizing patient views [30, 35]. Support from management and staff encouragement were seen as key in facilitating staff engagement with improvement related activities such as using and actioning PREM data [28, 31, 34, 35]. Staff reported that management allowing dedicated time to discuss PREM data, initiate improvements to health services and keeping staff informed about the use of patient feedback were key factors for successful PREM implementation [28, 29, 31, 36]. Furthermore, patient involvement in identifying areas for improvement [33, 37] could benefit implementation. Successful quality improvement programs require: (1) training and educational campaigns aimed at promoting quality improvement programs, (2) desire to deliver high quality patient-centered care by senior management and promoting a patient-centered culture within the hospital, (3) staff prompting and reminding each other to distribute PREMs and maintain data collection rates, (4) repeating surveys at regular intervals for longitudinal comparisons, (5) using a team-based approach to collect PREMs, and (6) making PREMs data visible and easily accessible to staff [4, 22, 28, 30, 31, 33, 35]. Staff reported that publicly releasing individual hospital PREM data for comparison across hospitals helped raise awareness of quality of care within institutions and its staff [28, 29].

#### Barriers to PREM implementation

Despite support for PREM usage in hospitals for health service evaluation and quality improvement, six studies reported several barriers related to hospital resources and environment [28, 30, 33, 34]. The most common barrier was resource limitations (e.g., availability of staff to collect and action PREM data) [28, 34, 35]. PREM completion is an additional responsibility for staff and time and resource constraints may adversely affect timely administration, consequently reducing the number of PREMs implemented for quality improvement purposes[28, 34]. Insufficient funding for PREM collection infrastructure prevented some hospitals from collecting PREM data or being able to prioritize and implement changes needed to achieve significant improvements to care [28, 30]. Reluctance among staff to overburden patients with paperwork by administering surveys during discharge [26, 28, 29, 33] may also influence implementation. The perception that patients would be overburdened with paperwork could limit the opportunity for patients to provide feedback on their experiences [26, 28, 29, 35]. Additional barriers noted by staff included: (1) lack of commitment to quality improvement, (2) worry about being held accountable for poor service outcomes, and (3) uncertainty about how to use PREMs data [30, 31, 35]. Staff also reported the collection of PREMs led to considerably increased workload due to the high volume of patients seen at healthcare services [30, 31, 35]. Although hospitals valued patient’s perspectives and established clear objectives about the importance of quality improvement, these barriers prevented staff from spending the time necessary to engage in quality improvement-related activities and initiatives. Additional staff-related barriers included skepticism about the usefulness of PREM data, lack of awareness about the importance of quality improvement programs, lack of interest in collecting PREM data, and unwillingness to change, improve or participate in quality improvement initiatives [31, 35]. Some staff also questioned whether they had the skills necessary to improve patient-centered care [4, 35]. Other staff challenges included concerns about the technology or automated data systems used for PREM collection as well as difficulties analyzing, interpreting and translating findings into actionable information [33, 35].

#### Healthcare providers’ perspectives towards PREMs

Studies that explored staff perspectives to using PREMs routinely in practice reported contradictory views [22, 23, 25, 34]. Some studies reported a positive outlook to PREMs, reporting the information gathered was invaluable, positively impacted their work and provided additional motivation to improve their skills, while others found staff felt less certain about the usefulness of PREMs [22, 25]. The main concern was patients’ ability to provide accurate and relevant feedback; mainly due to positive bias (patients tendency to provide positive feedback and reluctance to criticize their healthcare provider) and halo effects (patients’ tendency to attribute a previous negative experience to future healthcare experiences) [4, 22, 31]. Other factors reported to impact patients’ ability to provide accurate feedback included failure to understand experience measures, inconsistency between different patients despite similar experiences, and inability to evaluate clinical competence [4, 22, 31].

Overall, healthcare providers’ reported PREMs data could be used to prevent or minimize harm to patients by reducing risk of injury [29, 33] as well as providing information important for an institution’s safety practices and process [21, 26, 35]. PREM data was also useful for improving staff awareness of the quality of healthcare services provided and identifying areas needing improvement that staff were not aware of at both an individual and organizational level [21, 29, 33, 37]. PREM feedback, particularly when positive, served as an important opportunity to commend staff and reinforce good practice [4, 35, 37]. However, some staff stated that compliments were not treated in the same way as complaints. It was reported that complaints about staff were given high importance, prompting immediate action but compliments were not celebrated. Some staff considered quantitative data to be of limited value in understanding patient experiences and favored qualitative data (in the form of patient narratives and quotes), which were considered to facilitate more in depth understanding of patient concerns and experiences [22, 29, 31].

#### Patients’ perspectives towards PREMs

Three studies exploring patients’ perspectives on using PREMs routinely in practice reported the biggest challenge patients faced in providing feedback was the fear of being questioned about their treatment process and asked for an opinion about it while they were receiving treatment [24, 31, 32]. Patients stated that PREMs data have potential to improve overall care coordination and provided a unique opportunity to produce timely feedback on measurable processes and outcomes so that healthcare teams could use PREM data to improve their overall performance [24, 32]. Patients also reported an implementation challenge to be addressed by hospitals was to identify competent staff to help sustain the quality improvement program [24, 32].

#### Advantages of PREMs

PREMs assessing quality improvement focused on factors such as physician and nursing care, staff courtesy, cleanliness, comfort, waiting times, education about follow-up after discharge as well as satisfaction with information provided, the hospital system, services (e.g. food) and treatment outcomes such as pain management [21, 22, 26, 28, 30]. Some health services combined staff (clinicians, allied health, nurses) opinions with PREM data, recognizing the link between personal, professional and team development [21, 39]. Notable advantages of using PREMs in hospitals/clinics included: (1) gaining insight into patient perspectives, (2) longitudinal data to guide the development and implementation of quality improvement activities, monitoring changes and sustainability of improvement efforts, (3) early identification of new areas for quality improvement, (4) helping management prioritize action plans necessary to improve the quality of patient-centred care, and (5) offering management and staff the opportunity to evaluate their efforts to improve health services [8, 21–23, 26, 28, 30, 35, 37, 39]. Additionally, PREMs enabled comparison across clinicians, practices, and health services, and may motivate individual hospitals towards quality improvement [8, 21–23, 26, 28, 30, 35, 37, 39]. For patients, completing PREMs at regular intervals may help track their experiences in health services. PREMs that included a section for free text comment helped patients describe their expectations from their clinician and note any questions they wanted to ask clinicians during their consultations [8, 28, 31, 34].

#### Limitations and practical considerations to reduce resistance to PREM usage

Staff reported PREM data from a single patient may not detect key areas for service level quality improvement. Rather, PREM data should be aggregated at sufficient intervals (e.g., three monthly) to enable key areas for improvement to be detected [27, 28, 35, 37]. However, some clinicians felt the cost of collecting PREMs in a large sample was not worth the information generated. Others worried that collecting and reporting PREM data might be perceived as a threat to health service providers [22, 37]. Communication skills training for clinical and nonclinical staff to facilitate PREM-related conversations with patients might encourage staff to administer PREMs [23, 27, 28, 34, 35]. Staff also reported PREMs could be time consuming for patients to complete and raised expectations that could not be met by hospitals or individual staff [34]. Some patients worried about confidentiality and anonymity of their PREM data and how it would be used [4, 34].

Studies reported that in order to reduce staff resistance to using PREMs routinely in clinical practice, it was important to provide: training on the value of PREMs, clear guidance on data collection to avoid involuntary errors, assistance to choose the right measures for the context and intended purpose (e.g., appropriate for the particular medical condition/diagnosis), and discussion of results relevant to improving health services [31, 35, 37, 39, 40].

## Discussion

This research systematically reviewed the qualitative literature on patients’ and healthcare providers' perspectives towards using PREMs in routine clinical care. We identified six main themes reflecting barriers and facilitators to PREM implementation, patients’ and healthcare providers’ perspectives towards using PREMs, advantages of using PREMs in clinical practice, and limitations and practical considerations to reduce resistance to PREM usage.

Key facilitators to PREM implementation were organizational culture, support from management, dedicating time to PREM activities in hospitals, quality improvement programs, and providing comparisons of PREM data across hospitals. Key barriers to PREM implementation included resource constraints such as limited staff availability, lack of time, insufficient funding, reluctance and resistance among staff, lack of clarity on how to use PREM data, and concerns about technology used to process PREM data.

Barriers identified were broadly similar to previous literature: lack of resources [28, 34, 35], time, and expertise in analysis of data and quality improvement [35, 40]. Another barrier commonly reported was lack of training for staff, suggesting that staff were interested in better understanding of how to use and interpret PREM data [10, 26, 31, 35, 36]. In order to successfully shift towards a more patient-centered healthcare service, it is crucial to engage relevant staff (both clinical and nonclinical) in structured training across healthcare settings and create awareness about the importance and value of collecting PREM data [10, 23, 27, 28, 31, 34–36].

Implementing PREM data collection was challenging for some staff regardless of setting [28, 34, 35]. Data collection planning, persistence, and commitment from the management and staff were required [10, 11] to succeed in implementation. Clinical staff (doctors, nurses and allied health professionals) reported the importance of having a designated person to take ownership of data collection [29, 34] including identifying eligible patients, monitoring their enrollment and appointments, and contacting patients who did not return completed PREMs. These tasks demand additional weekly resources and were more challenging at clinics or hospitals that used paper-based medical records.

Clinical staff also noted that some patients’ dissatisfaction may be beyond their control. This includes patients assuming newer devices and interventions had better care standards and were disappointed to find older technology used in hospitals. Similarly, patients with multiple comorbid conditions such as diabetes, hypertension, and substance abuse may experience worsening symptoms during their treatment or procedures, and this can be unsatisfactory for patients; individuals have varying tolerance for pain and this may impact their overall care experience; or insurer limitations may result in patients’ reporting unsatisfactory experiences related to their healthcare service [29, 34].

Healthcare providers’ perspectives towards using PREMs were mixed. Some reported PREM data improved services and awareness among staff about the services they provided. Collecting and analyzing PREM data can improve patients’ experiences and transform practices and medical institutions [41]. However, other healthcare providers reported additional concerns about accuracy of patient feedback because several other factors such as positive bias, halo effect, timing of survey administration, and differing expectations may influence patients’ perceptions of the services they receive. These results are further supported by previous studies [42–44] which report patient perceptions about the quality of care received correlated with lower complication rates and clinical quality of the medical institution (as measured by performance indicators). Further, Stein et al. [44] reported that some healthcare providers felt patient satisfaction was a poor indicator of quality of care.

Patient experiences and outcomes may also be confounded by characteristics such as age, disease stage and phase, in the context of chronic or acute illness [45]. Therefore, to ensure patient feedback and data add value to organizations, they may be integrated with indicators from other sources (such as their treatment and related side effects, insurance experience, comorbid conditions, wait times, logistics, and any other inconveniences). Taking a multi-dimensional performance evaluation approach would facilitate implementation of PREMs in routine care, and staff may not always be held responsible for patient dissatisfaction. Furthermore, integrated data may help organizations understand the underlying relationship between effectiveness, safety, and experience at the patient level.

Consistent with others [46], we found that patients may be burdened with paperwork during discharge, thus limiting the opportunity for them to provide accurate feedback on their experiences. To reduce this burden, web based PREMs can be introduced, allowing patients to complete them at home post-discharge. This methodology has been proven to be inexpensive for organizations and timesaving for staff, when compared to postal surveys or telephone surveys [47] and does not cause bias in the results [48].

This review supports the positive impact of care coordination on patients’ experience and found that patients considered PREMs a critical feedback tool to assess and improve the quality of care across various clinical settings and between staff within a healthcare team [24, 32]. Arguably, for some health conditions such as chronic wounds, disease specific PREMs may be useful to assess condition-specific aspects of care coordination, interdisciplinary communication, and shared decision-making [24, 32].

Longitudinal data collected using PREMs may also enable patient participation in articulating and mapping their own experiences of health services. Despite challenges and limitations, service providers who persevered with data collection, put in efforts to strengthen patient relationships, and engaged patients in the assessment and planning process capitalized on the benefits of PREMs data [28, 34].

Findings from this review highlight clinicians’ concerns about collecting PREMs, particularly their worries about disruption of patient care [8, 28, 35, 38]. Frequent feedback was perceived as more useful than only annual data collection for quality improvement initiatives. However, some were skeptical about the validity of results [26, 28, 34]. Further, patients appreciated prompt recognition of their complaints and feedback [29, 34] and were more likely to complete PREMs if they perceived their feedback led to improvements in their health service experience.

Hence, to ensure quality improvement programs are successful, hospitals should take patients’ feedback into immediate consideration and make efforts to improve their experience [29, 34]. For patients to complete PREMs successfully, it is essential to train staff on why and how to use PREMs within the context of their specific clinical environment. For example, inviting patients to complete PREMs on a busy clinic day may result in lower completion rates compared to collection of PREMs on a quiet day [8, 26, 29, 33]. Including real-time feedback systems into web based PREMs to instantly capture patients’ feedback and to provide quick response to any issues reported could promote a patient-driven quality improvement culture. This may be well received by patients when done regularly. Web based PREMs would facilitate PREM data collection, but requires investment in set-up of software systems for data collection and processing.

To our knowledge this is the first review to synthesize the qualitative evidence on healthcare professional and patient perspectives and experiences of using PREMs in clinical practice. This helps guide future quality improvement initiatives. However, limitations of this review should be considered when interpreting the findings. Overall, quality of study design and conduct for some studies was unclear due to the poor quality of reporting. Quality improvement initiatives using PREMs may occur within hospitals and clinics regularly, but findings may be held locally and not publicly available. Further, this review only included articles published in English so the experiences of using PREMs in countries where English is not the first language were not represented. Nevertheless, a range of common themes were identified across studies, providing useful information for future PREMs related research.

Some notable gaps were identified in this review. Only four studies explored patients’ experiences and perspectives of PREMs. We did not extract PREM development and psychometric properties as this was beyond the scope of this paper. Consequently, we excluded studies focused on reporting PREM development, validation, or selection for use in a particular clinical setting. Future research should explore this and how PREMs data are currently being used in everyday clinical practice and describe any structured plans developed by hospitals or healthcare organizations on how to use patient feedback for quality improvement. Understanding patient perceptions of using PREMS in routine clinical care is critical to optimize patient engagement and may potentially alleviate staff concerns about overburdening patients. In the context of healthcare improvements, further investigation is needed into how PREM data is used to inform changes to the delivery of health services and provider behavior. Future PREMs initiatives should also include follow-up assessments to examine changes in patient experiences because of improvements made by hospitals based on feedback received.

## Conclusions

This study highlights the advantages of collecting and using PREM data from the healthcare professionals’ and patients’ perspectives and identifies barriers and facilitators to implementing PREMs into routine clinical care. The primary use of PREMs has been to initiate health service quality improvements. Key factors facilitating and hindering the collection and use of PREMs include organizational-, staff- and patient-related factors. Sufficient resources, support from organization leadership, formal staff training in using and interpreting PREM data, choosing the right measure, and patient engagement in the assessment and planning are crucial for the benefits of PREM data to be realized. Future studies may focus on addressing barriers to PREM implementation and evaluate the effects of PREMs on health service quality improvements.

## Supplementary Information

**Additional file 1.** Search strategy.

## Data Availability

Results from the searches used and/or analyzed during the current study are available from the corresponding author on reasonable request.

## References

[1] Bull C (2019). A systematic review of the validity and reliability of patient-reported experience measures. Health Serv Res.

[2] Hodson M, Andrew S (2014). Capturing experiences of patients living with COPD. Nurs Times.

[3] Male L (2017). Measuring patient experience: a systematic review to evaluate psychometric properties of patient reported experience measures (PREMs) for emergency care service provision. Int J Qual Health Care.

[4] Burt J (2017). Improving patient experience in primary care: a multimethod programme of research on the measurement and improvement of patient experience. Programme Grants Appl Res.

[5] Intelligence S (2014). Evaluation of the NHS Institute patient experience learning programme.

[6] Antunes B (2014). Implementing patient-reported outcome measures in palliative care clinical practice: a systematic review of facilitators and barriers. Palliat Med.

[7] Bastemeijer CM (2019). Patient experiences: a systematic review of quality improvement interventions in a hospital setting. Patient Relat Outcome Meas.

[8] Davies E, Cleary PD (2005). Hearing the patient’s voice? Factors affecting the use of patient survey data in quality improvement. BMJ Qual Saf.

[9] Doyle C, Lennox L, Bell D (2013). A systematic review of evidence on the links between patient experience and clinical safety and effectiveness. BMJ Open.

[10] Gleeson H (2016). Systematic review of approaches to using patient experience data for quality improvement in healthcare settings. BMJ Open.

[11] Groene O (2011). Patient centredness and quality improvement efforts in hospitals: rationale, measurement, implementation. Int J Qual Health Care.

[12] Tricco AC (2014). Safety, effectiveness, and cost effectiveness of long acting versus intermediate acting insulin for patients with type 1 diabetes: systematic review and network meta-analysis. BMJ.

[13] Coulter A, Fitzpatrick R, Cornwell J (2009). Measures of patients' experience in hospital: purpose, methods and uses.

[14] Robert G, Cornwell J (2013). Rethinking policy approaches to measuring and improving patient experience.

[15] Byron SC (2014). Developing measures for pediatric quality: methods and experiences of the CHIPRA pediatric quality measures program grantees. Acad Pediatr.

[16] The Health Foundation (2011). Are clinicians engaged in quality improvement? A review of the literature on healthcare professionals’ views on quality improvement initiatives, in The Healthcare Foundation Inspiring Improvement.

[17] Moher D (2009). Preferred reporting items for systematic reviews and meta-analyses: the PRISMA statement. PLoS Med.

[18] McDonagh M, Peterson K, Raina P et al (2013) Avoiding bias in selecting studies. In: Methods guide for effectiveness and comparative effectiveness reviews [Internet]. Agency for Healthcare Research and Quality (US), Rockville, MD. Available from: https://www.ncbi.nlm.nih.gov/books/NBK126701/23487864

[19] Tong A, Sainsbury P, Craig J (2007). Consolidated criteria for reporting qualitative research (COREQ): a 32-item checklist for interviews and focus groups. Int J Qual Health Care.

[20] Thomas J, Harden A (2008). Methods for the thematic synthesis of qualitative research in systematic reviews. BMC Med Res Methodol.

[21] Carter M et al (2004) Impact of systematic patient feedback on general practices, staff, patients and primary care trusts. Educ Prim Care 15(1):30–38

[22] Farrington C (2017). Doctors’ engagements with patient experience surveys in primary and secondary care: a qualitative study. Health Expect.

[23] Friedberg MW (2011). Physician groups’ use of data from patient experience surveys. J Gen Intern Med.

[24] Siantz E, Henwood B, Gilmer T (2020). Patient experience with a large-scale integrated behavioral health and primary care initiative: a qualitative study. Fam Syst Health.

[25] Boyce MB, Browne JP, Greenhalgh J (2014). Surgeon’s experiences of receiving peer benchmarked feedback using patient-reported outcome measures: a qualitative study. Implement Sci.

[26] D’Lima D (2017). Continuous monitoring and feedback of quality of recovery indicators for anaesthetists: a qualitative investigation of reported effects on professional behaviour. BJA Br J Anaesth.

[27] Davies E (2008). Evaluating the use of a modified CAHPS® survey to support improvements in patient-centred care: lessons from a quality improvement collaborative. Health Expect.

[28] Reeves R, Seccombe I (2008). Do patient surveys work? The influence of a national survey programme on local quality-improvement initiatives. BMJ Qual Saf.

[29] Scott J (2019). Implementing a survey for patients to provide safety experience feedback following a care transition: a feasibility study. BMC Health Serv Res.

[30] Barr JK (2006). Using public reports of patient satisfaction for hospital quality improvement. Health Services Res.

[31] Berger S, Saut AM, Berssaneti FT (2020). Using patient feedback to drive quality improvement in hospitals: a qualitative study. BMJ Open.

[32] Squitieri L (2020). Patient-reported experience measures are essential to improving quality of care for chronic wounds: an international qualitative study. Int Wound J.

[33] Lucock M (2015). A mixed-method investigation of patient monitoring and enhanced feedback in routine practice: barriers and facilitators. Psychother Res.

[34] van Rooijen M (2020). Implementation of a Patient Reported Experience Measure in a Dutch disability care organisation: a qualitative study. J Patient Rep Outcomes.

[35] Davies EA (2011). Factors affecting the use of patient survey data for quality improvement in the Veterans Health Administration. BMC Health Serv Res.

[36] Alvarado N (2020). Exploring variation in the use of feedback from national clinical audits: a realist investigation. BMC Health Serv Res.

[37] Tirado AC (2017). Using patient-reported outcome measures for quality improvement in clinical genetics: an exploratory study. J Genet Couns.

[38] Heinemann AW (2017). Enhancing quality of prosthetic services with process and outcome information. Prosthet Orthot Int.

[39] Duncan EA, Murray J (2012). The barriers and facilitators to routine outcome measurement by allied health professionals in practice: a systematic review. BMC Health Serv Res.

[40] Philpot LM (2018). Barriers and benefits to the use of patient-reported outcome measures in routine clinical care: a qualitative study. Am J Med Qual.

[41] Browne K (2010). Analysis & commentary measuring patient experience as a strategy for improving primary care. Health Aff.

[42] Glenngård AH, Anell A (2017). Does increased standardisation in health care mean less responsiveness towards individual patients’ expectations? A register-based study in Swedish primary care. Sage Open Med.

[43] Glenngård AH, Anell A (2018). Process measures or patient reported experience measures (PREMs) for comparing performance across providers? A study of measures related to access and continuity in Swedish primary care. Prim Health Care Res Dev.

[44] Stein SM (2015). Patients’ perceptions of care are associated with quality of hospital care: a survey of 4605 hospitals. Am J Med Qual.

[45] Elliott MN (2013). Care Experiences of managed care M edicare enrollees near the end of life. J Am Geriatr Soc.

[46] De Brún A (2017). PR eSaFe: a model of barriers and facilitators to patients providing feedback on experiences of safety. Health Expect.

[47] Blood Z (2021). Implementation of patient-reported outcome measures and patient-reported experience measures in melanoma clinical quality registries: a systematic review. BMJ Open.

[48] Rutherford C (2016). Mode of administration does not cause bias in patient-reported outcome results: a meta-analysis. Qual Life Res.

