# Supplementary material for: Patient and healthcare provider perceptions on using patient-reported experience measures (PREMs) in routine clinical care: a systematic review of qualitative studies
Source: J Patient Rep Outcomes. 2022 Dec 2;6:122. doi: 10.1186/s41687-022-00524-0 (PMC9718906; doi:10.1186/s41687-022-00524-0)
Supplement: Supplementary file 1 — Additional file 1. Search strategy. [file 41687_2022_524_MOESM1_ESM.docx]

# SEARCH STRATEGY

1. (patient adj reported adj (experience$ or outcome$ or data)).ti,ab,kw.

2. (patient adj based adj measure$).ti,ab.

3. (PRO or PROM or PREM).ti,ab.

4. ((quality adj1 life) or HRQ$L or QOL).ti,ab,kw.

5. symptom report$.ti,ab.

6. (patient adj (report* or feedback)).ti,ab.

7. feedback.ti.

8. or/1-7

9. (routine adj (practice or administration or collection)).ti,ab.

10. (health$care adj (provider or professional or facility or setting or context)).ti,ab,kw.

11. ((service or health$care) adj (quality or evaluation)).ti,ab,kw.

12. quality adj (improvement or assessment or evaluation).ti,ab,kw.

13. (clinic or clinics or routine).ti.

14. or/9-13

15. qualitative.ti,ab,kw,pt.

16. interview$.ti,ab.

17. (focus group$).ti,ab

18. or/15-17

1. 8 and 14 and 18
